# Inhibition of Phosphoinositide 3-Kinase/Protein Kinase B Signaling Hampers the Vasopressin-dependent Stimulation of Myogenic Differentiation

**DOI:** 10.3390/ijms20174188

**Published:** 2019-08-27

**Authors:** Silvia Sorrentino, Alessandra Barbiera, Gabriella Proietti, Gigliola Sica, Sergio Adamo, Bianca Maria Scicchitano

**Affiliations:** 1 Istituto di Istologia ed Embriologia, Università Cattolica del Sacro Cuore, Fondazione Policlinico Universitario A. Gemelli IRCCS, 00168 Roma, Italy; 2 Dipartimento di Scienze Anatomiche, Istologiche, Medico-legali e dell’Apparato Locomotore (SAIMLAL), Sezione di Istologia ed Embriologia Medica, Sapienza Università, via A. Scarpa 16, 00161 Roma, Italy

**Keywords:** skeletal muscle, LY294002, b-HLH-MRFs, HDACs, mTOR, CaMK

## Abstract

Arginine-vasopressin (AVP) promotes muscle differentiation, hypertrophy, and regeneration through the combined activation of the calcineurin and Calcium/Calmodulin-dependent Protein Kinase (CaMK) pathways. The AVP system is impaired in several neuromuscular diseases, suggesting that AVP may act as a physiological factor in skeletal muscle. Since the Phosphoinositide 3-kinases/Protein Kinase B/mammalian Target Of Rapamycin (PI3K/Akt/mTOR) signaling plays a significant role in regulating muscle mass, we evaluated its role in the AVP myogenic effect. In L6 cells AKT1 expression was knocked down, and the AVP-dependent expression of mTOR and Forkhead box O3 (FoxO) was analyzed by Western blotting. The effect of the PI3K inhibitor LY294002 was evaluated by cellular and molecular techniques. Akt knockdown hampered the AVP-dependent mTOR expression while increased the levels of FoxO transcription factor. LY294002 treatment inhibited the AVP-dependent expression of Myocyte Enhancer Factor-2 (MEF2) and myogenin and prevented the nuclear translocation of MEF2. LY294002 also repressed the AVP-dependent nuclear export of histone deacetylase 4 (HDAC4) interfering with the formation of multifactorial complexes on the myogenin promoter. We demonstrate that the PI3K/Akt pathway is essential for the full myogenic effect of AVP and that, by targeting this pathway, one may highlight novel strategies to counteract muscle wasting in aging or neuromuscular disorders.

## 1. Background

Myogenesis is a coordinated sequence of events whereby myoblasts differentiate into myotubes, irreversibly exit the cell cycle, and undergo profound structural and functional modifications. Factors belonging to the b-HLH DNA-binding superfamily regulate the transcription of muscle-specific genes. In addition to myogenin, these include the redundant MRFs Myf-5, MyoD, and MRF4 [1,2]. While Myf5 regulates the proliferation rate of myoblasts, and MyoD is required for their differentiation [3,4], the appearance of myogenin reflects the irreversible withdrawal of myoblasts from the cell cycle and, upon the simultaneous presence of MRF4, their terminal differentiation [3,5,6]. Basic-HLH-MRFs heterodimerize with E2A gene products (E12/E47), thus binding the E-box consensus sequences (CANNTG) in the promoter regions of muscle-specific genes, inducing their transcription [7]. The MEF2-family transcription factors function as accessory regulators of muscle gene expression and differentiation since they bind to and cooperate with b-HLH-MRFs to synergically induce muscle-specific genes [8,9,10].

A large body of evidence indicates that b-HLH-MRFs and MEF2 activities are modulated by epigenetic modifications by which chromatin-modifying enzymes and remodeling complexes reprogram muscle promoters at various stages, finely tuning gene expression [11,12]. In particular, HDACs and histone acetyltransferases (HATs) alter chromatin structure through post-translational modifications, thus regulating the transcriptional activity of specific genes [13]. Members of class II HDACs (HDACs 4-5-7), expressed in skeletal muscle cells, interact with MEF2 and specifically inhibit myoblast differentiation by repressing MEF2 activity at the level of muscle-specific promoters [14,15,16,17,18]. HDAC 4-5-7 proteins shuttle from the nucleus to the cytoplasm upon differentiation of myoblasts into post-mitotic myotubes [14,15,16,18]. Therefore, the nuclear-cytoplasmic trafficking of class II HDACs provides an appealing mechanism for the regulation of the activity of muscle transcription factors.

The genetic and epigenetic regulatory mechanisms of myogenesis outlined above are exploited by the neurohypophyseal hormones AVP, oxytocin (OT), and related peptides, in their non-canonical role as potent inducers of myogenic differentiation in both in vitro and in vivo experimental models [3,9,18,19,20,21,22,23]. Indeed, we have previously shown that AVP induces myogenesis through a mechanism involving the transcriptional activation of myogenin at the MEF2 site of the myogenin promoter. We have also demonstrated that the AVP-dependent myogenic differentiation involves the activation of the Ca^2+^/calmodulin-dependent protein kinase (CaMK) and the Calcineurin A (CnA) signaling pathways [9,18,20,21,22,23]. The AVP-dependent combined activation of both pathways results in the formation of multifactorial complexes on the promoters of muscle-specific genes and strongly stimulates myogenic differentiation [9,18,20,21,22].

Neurohypophyseal hormone receptors are present in myogenic cells or skeletal muscle [20,24,25], and it has been demonstrated that the expression of the V1a AVP-receptor (V1aR) is modulated during skeletal muscle regeneration [21]. The V1aR is a 7-transmembrane domain-G-protein coupled receptor, whose occupancy activates phosphoinositide 3-kinases (PI3Ks) [26]. Receptor-mediated stimulation of PI3K induces phospholipase C (PLC) activation, which triggers a downstream signaling cascade leading to the formation of inositol triphosphate, diacylglycerol and the activation of Protein Kinase C (PKC) [27,28,29,30]. This cascade appears particularly complex in the light of an elaborate mutual regulation between PLC and PI3K [31]. Numerous studies demonstrated the role of PI3Ks in the regulation of cell metabolism, proliferation, and survival. The vast array of such effects depends on the wide range of molecules possessing 3-phosphoinositide-binding domains [26,32]. The pleckstrin homology (PH) domain is one of such domains, present on a wide range of downstream targets of PI3K, including the protein kinase B/Akt [33,34] known to be involved in the regulation of cell survival and proliferation.

The PI3K/Akt signaling plays a significant role in the regulation of skeletal muscle mass controlling both protein synthesis and degradation [35,36,37]. Indeed, Akt controls the fiber protein homeostasis in two ways: on the one hand it indirectly enhances the activity of mTOR which, in turn, promotes protein synthesis through the S6 kinase-dependent phosphorylation of the ribosomal protein S6; on the other hand Akt phosphorylates the FoxO transcription factors, thus repressing their activity and resulting in the inhibition of protein degradation. A reduction in the activity of the Akt pathway results in the FoxO-dependent transcriptional regulation of the ubiquitin ligase atrogin-1, leading to increased protein catabolism [38,39,40].

We previously demonstrated that the stimulation of AVP-V1aR signaling, in both in vivo and in vitro experimental models, results in a substantial enhancement of muscle regeneration, favoring fiber hypertrophy, and counteracting Tumor Necrosis Factor (TNF)-induced muscle atrophy [22,23,41]. A key role in the myogenic effect of AVP is exerted by the stimulation of the calcineurin and CaMK signaling pathways. Furthermore, the hypertrophying effect of AVP on cultured myogenic cells and during muscle regeneration [18,19,21] is consistent with the activation of Akt downstream of PI3K.

Since, as mentioned above, CaMK signaling plays a crucial role in the myogenic effect of AVP and since it is known that in several systems, such as normal and neoplastic B-lymphoid cells, the Akt/mTOR signaling is stimulated by the Ca^2+^-dependent activation of the CaMK pathway [42,43], we speculate the possibility of and additional indirect link between Akt/mTOR pathway and the AVP signaling through CaMKs stimulation. Moreover, the role of AVP as a physiological factor in skeletal muscle homeostasis is also suggested by evidence showing that the AVP system is impaired in several neuromuscular diseases such as amyotrophic lateral sclerosis and multiple sclerosis [44,45,46].

Here, for the first time, we demonstrate that the impairment of the PI3K/Akt signaling by genetic and pharmacological approach results in mTOR, MEF2 and myogenin downregulation, affects the cellular localization of MEF2 preventing its nuclear translocation, upregulates the expression of FoxO3a leading to the activation of ubiquitin ligase atrogin-1, thus impinging upon the AVP-dependent myogenic differentiation.

## 2. Results

### 2.1. LY294002 Inhibits the AVP-Dependent Myogenic Differentiation of L6 Cells

**Figure 1:**
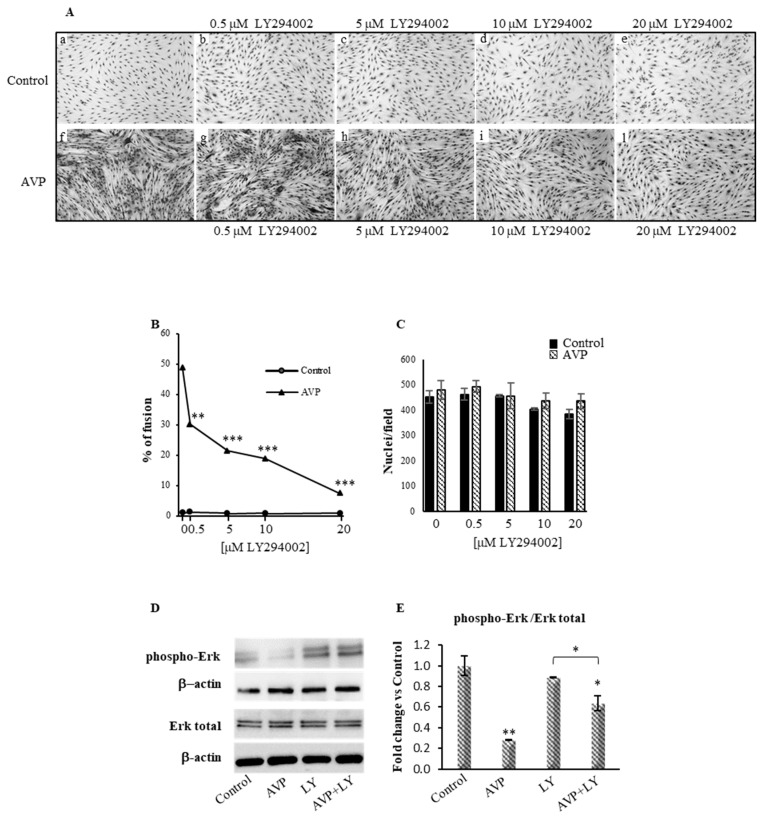
Effects of LY294002 on the vasopressin (AVP)-dependent myogenic differentiation. (**A**) Morphological analysis of L6 cells cultured in growth medium, shifted in serum-free medium after 24 h and treated with increasing concentrations of LY294002 (0, 0.5, 5,10 and 20 μM) in the absence (**a**–**e**) or in the presence of 0.1 μM AVP (**f**–**l**). After a 6-day treatment, morphological differentiation was evaluated by May-Grünwald Giemsa staining (original magnification, 10×). (**B**) In similarly prepared cultures of L6 cells, nuclei were counted under the microscope and the ratio between the number of nuclei in myotubes versus the total number of nuclei per microscopic field, in both LY294002 and AVP + LY294002 treated cells, were expressed as the percentage of fusion and (**C**) as the total (fused and unfused) nuclei/microscopic field. Each experimental point represented in the graphs is the mean ± SD of the counts evaluated from three to five independent samples obtained in three separate experiments. (**D**) Western blot analysis of phospho-p44/42 and total p44/42 expression performed after 48 h of culture. (**E**) Densitometric analysis was achieved using an anti-β-actin antibody to verify the equal loading of the samples. Statistical analysis was performed using Student’s *t*-test. * *p* < 0.05; **p < 0.01; *** *p* < 0.001. For Figure 1B * vs. AVP; for Figure 1F # vs. AVP; for Figure 1E * vs. Control and ^#^ vs. LY294002.

In order to investigate the role of PI3K in mediating the action of AVP on myogenic cells, we used a well-characterized serum-free culture condition, which allows studying the mechanism of action of the neurohypophyseal hormones in the absence of confounding effects of serum factors [9,19]. In this condition, while the L6 cells cultured in the absence of AVP (Control, Figure 1Aa) underwent virtually no fusion, the addition of 0.1 µM AVP strongly stimulated L6 cell fusion (Figure 1Af,B) without evident effects of AVP on total nuclei (Figure 1C). This effect is in agreement with our previous results [19] and with the expression of the phosphorylated p44/42 (Erk1/2), a marker of cell proliferation, which was normalized with both total p44/42 and β-actin (Figure 1D,E).

The presence of increasing concentrations of LY294002 (0.5–20 µM) did not significantly affect either the cell density of control and AVP-treated cells or the extremely modest fusion level of control cells, but significantly and dose-dependently inhibited the AVP-dependent fusion of L6 cells (Figure 1Ab–e,g–l). In fact, in the presence of 20 µM LY294002, the AVP-dependent fusion of L6 cells was completely inhibited (Figure 1B). Superimposable results were obtained measuring the activity of CK [19,47] in extracts of L6 cells cultured as described above (6.6-fold stimulation of CK specific activity in AVP-treated compared to control cells; complete inhibition of the effect of AVP at 20 µM LY294002: not shown).

### 2.2. LY294002 Counteracts the AVP-Dependent Stimulation of PI3K/Akt/mTOR Signaling

The PI3K/Akt/mTOR signaling plays a leading role in the regulation of skeletal muscle mass [35,37]. Akt inhibits protein degradation by phosphorylating, and thus repressing, the transcription factors of the FoxO family, and stimulates protein synthesis via mTOR [36,38,48].

**Figure 2:**
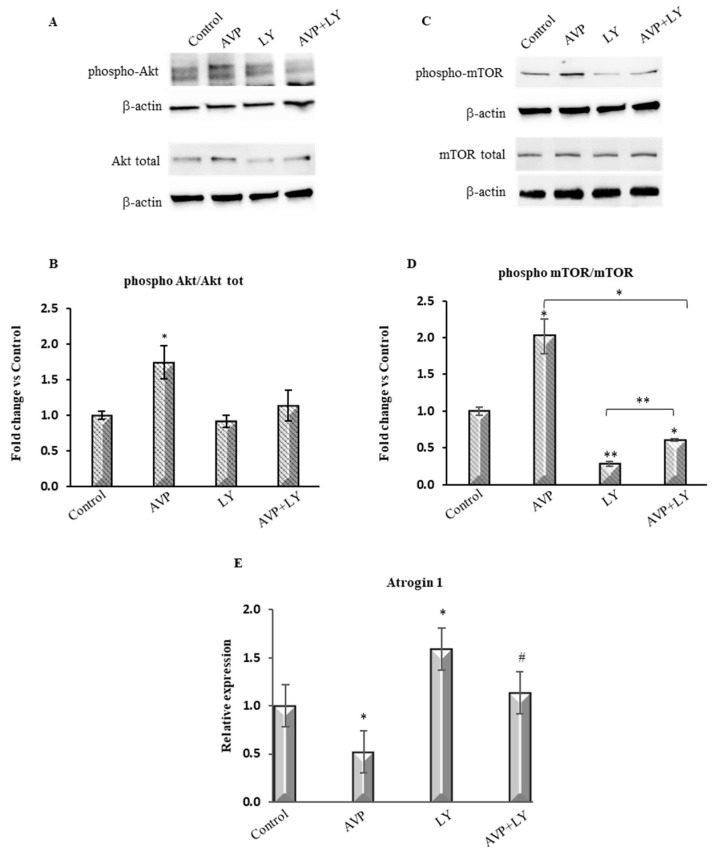
LY294002 inhibits the AVP-dependent stimulation of PI3K/Akt/mTOR pathway. L6 cells were plated in growth medium and after 24 h were shifted in serum-free medium and treated with 0.1 μM AVP in the presence or absence of 20 μM LY294002. (**A**) Western blot analysis of phospho-Akt and Akt total performed after 48 h of cultures. (**B**) Densitometric analysis was achieved using an anti-β-actin antibody to verify the equal loading of the samples (**C**) Western blot analysis of phospho-mTOR and mTOR total performed after 48 h of cultures. (**D**) Densitometric analyses were achieved using an anti-β-actin antibody to verify the equal loading of the samples (**E**) Real-Time PCR analysis of Atrogin-1 in L6 cells treated as described above. Statistical analysis was performed using Student’s *t*-test. *^,#^ *p* < 0.05; ** *p* < 0.01; * vs. Control; ^#^ vs. LY294002.

To better investigate whether LY294002 effectively inhibited the AVP-dependent stimulation of the PI3K downstream signals, we analyzed the expression of phospho-Akt and phospho-mTOR in L6 cells treated with AVP alone or in combination with LY294002 for 48 h. Western blot analysis (Figure 2A–D) showed that AVP enhanced the ratio of the expression levels of phospho-Akt and phospho-mTOR to the respective total isoforms, each one normalized for β-actin. The effect of AVP on phospho-Akt and phospho-mTOR expression is strongly hampered by LY294002, as demonstrated by the low levels of the expression of these factors when the cells were treated with both AVP and LY294002. Besides, real-time PCR analysis revealed that Atrogin-1, a key target of the FoxO-dependent activation of the ubiquitin-proteasome pathway, was strongly downregulated upon AVP stimulation. Conversely, LY294002 treatment significantly increased its expression both in the presence and in the absence of AVP (Figure 2E).

These results confirm the involvement not only of PI3K but also of its downstream activated effectors, phospho-Akt, and phospho-mTOR, demonstrating the activation of the canonical PI3K/Akt pathway in AVP-stimulated myogenic cells. Consistently, treatment with LY294002 resulted in the inhibition of two crucial regulators of this pathway, Akt and mTOR, and upregulated the expression of the muscle-specific ubiquitin ligase atrogin-1.

### 2.3. Akt Knockdown Hampers the Myogenic Effect of AVP in L6 Cells

**Figure 3:**
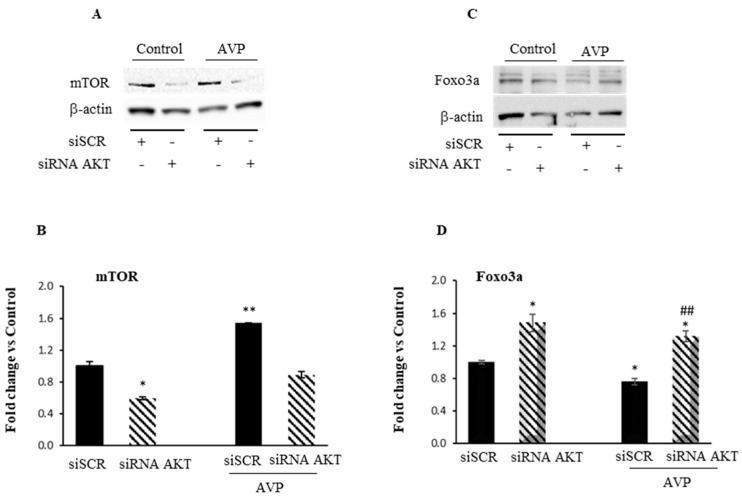
Akt knockdown hampered the myogenic effect of AVP in L6 cells. (**A**,**C**) Representative Western blots showing respectively mTOR and FoxO protein expression in L6 cells after silencing of AKT1. (**B**,**D**) Densitometric analyses were achieved using an anti-β-actin antibody to verify the equal loading of the samples. Statistical analysis was performed using Student’s *t*-test. * *p* < 0.05; **^,##^ *p* < 0.01; * vs. Control; ^#^ vs. AVP.

In order to confirm the involvement of PI3K in the AVP signaling, we used a genetic approach, i.d. AKT1 silencing, in addition to the pharmacological inhibition by LY294002. L6 cells were transiently transfected with AKT1 siRNA. After assessing the degree of Akt knockdown by Western blot analysis, and the transfection efficiency using the fluorescent transfection control DsiRNA (TYE 563) (see Supplemental Figure S1), we demonstrated that the downregulation of Akt leads to a substantial reduction of AVP-dependent mTOR expression (Figure 3A,B). Moreover, while after AVP treatment the FoxO expression level is significantly reduced compared to the control, its expression increases after AKT1 silencing even in the presence of AVP (Figure 3C,D). These findings corroborate our abovementioned results with PI3K/AKT pharmacological inhibition and demonstrate that AVP can control myogenesis, promoting the activity of mTOR on the one hand, and downregulating FoxO transcription factor, on the other.

### 2.4. LY294002-Dependent Inhibition of PI3K/Akt Signaling Hinders the AVP-Dependent Induction of Myogenin and MHC Expression

**Figure 4:**
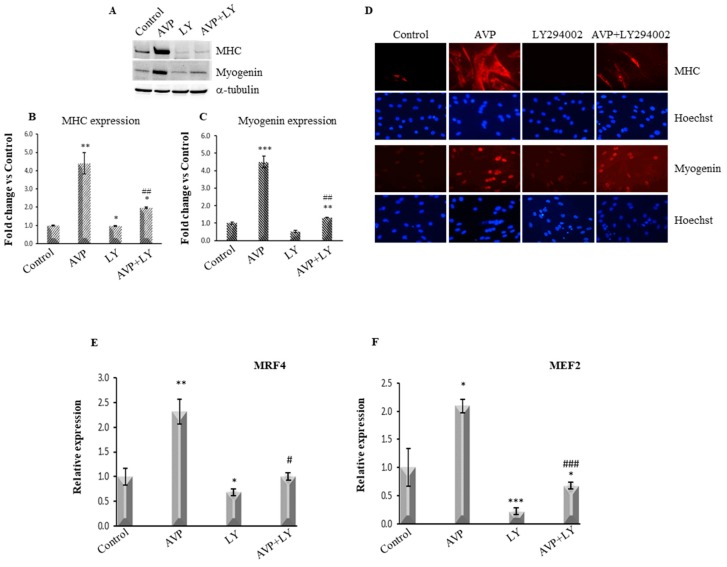
LY294002 down-regulates the AVP-dependent induction of MHC and myogenin expression. L6 cells were plated in growth medium and after 24 h were shifted in serum-free medium and treated with 0.1 μM AVP in the presence and in the absence of 20 μM LY294002. (**A**) Western Blot analysis of MHC and myogenin expression was performed after 48 h of culture. (**B**,**C**) Densitometric analyses were achieved using an α-tubulin antibody to verify equal loading of the samples. (**D**) Immunofluorescence analysis of L6 cells treated with the same condition described above was achieved to evaluate MHC and myogenin expression levels. (Magnification 20×). (**E**,**F**) Real-Time PCR analysis was carried out to evaluate the MRF4 (Myf6) and MEF2 mRNA expression levels. The experiments were performed three times, and representative results are shown. Statistical analysis was performed using Student’s *t*-test. *^,#^ *p* < 0.05; **^,##^ *p* < 0.01; ***^,###^ *p* < 0.001. * vs. Control; ^#^ vs. LY294002.

The expression and the activity of the b-HLH-MRFs are substantial determinants of skeletal muscle differentiation. Among the MRFs, myogenin controls the transcriptional activation of skeletal muscle structural genes such as Myosin heavy Chain (MHC) [5,6], regulating the transition from committed myoblasts to fully differentiated myotubes. Therefore, we analyzed the effects of PI3K/Akt signaling inhibition on both myogenin and MHC expression levels. L6 cells were cultured in serum-free medium and treated with 0.1 μM AVP alone or in combination with 20 μM LY294002 for 48 h (Figure 4A–D).

LY294002 treatment L6 cells resulted in a dramatic reduction of the expression of the AVP-stimulated levels of both myogenin and MHC, whereas very little or no effect of LY294002 was evident on the control levels of both myogenic markers, as shown by Western blotting assay (Figure 4A–C). We confirmed these data by immunofluorescence analysis. As shown in Figure 4D, LY294002 treatment dramatically downregulated the AVP-stimulated expression of both myogenin and MHC.

### 2.5. LY294002 Treatment Inhibits the AVP-Dependent Stimulation of MRF4 and MEF2 mRNA Expression

Basic HLH-MRFs regulate the myogenic program by cooperating with other more general factors such as the MEF2, which in turn regulate MRFs expression [13,49]. Among the MRFs, MRF4 (Myf6) differs from the other family members for a biphasic pattern of expression during mouse development [50] and is the predominant MRF expressed in adult muscle fibers [51]. MEF2 plays a crucial role in activating the expression of the MRF4 gene since normal MRF4 expression requires a proximal MEF2 site [52]. Our previous studies demonstrated the AVP role in the full expression of the myogenic program, an effect mediated by MEF2 [9,18]. Therefore, we investigated whether the inhibition of the PI3K/Akt signaling by LY294002 affects the AVP-dependent expression of MRF4 and MEF2. To this purpose, we analyzed by Real Time-PCR the expression levels of MRF4 and MEF2 in L6 cultures after 48 h of treatment with 0.1 μM AVP and 20 μM Ly294402, individually or in combination. As shown in Figure 4E,F, LY294002 treatment inhibited both the basal and the AVP-stimulated expression level of these factors.

### 2.6. LY294002 Hampers the AVP-Dependent Activation of the 84-bp Myogenin Promoter

**Figure 5:**
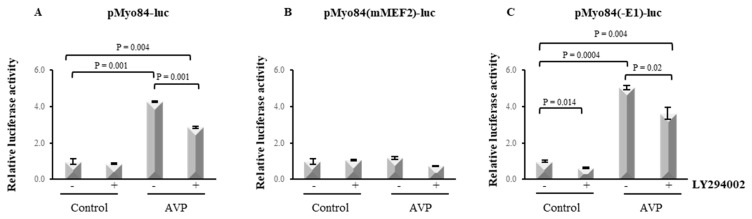
LY294002 inhibits the AVP-dependent activation of myogenin promoter through the MEF2 site. L6 cells were plated in growth medium and after 24 h were transiently transfected with (**A**) the luciferase reporter construct carrying the 84-bp sequence of the myogenin promoter carrying intact MEF2 and E-box sites or (**B**) with the same promoter mutated in the MEF2 site or (**C**) deleted of the E-box. After 24 additional hours, cultures were shifted to serum-free medium and treated with 0.1 μM AVP in the presence and in the absence of 20 μM LY294002 for 48 h. The luciferase activity was determined and normalized to β-gal activity as described in Materials and Methods. Statistical analysis was performed by Student’s *t*-test on data obtained from three independent experiments.

A MEF2 binding site is present in the promoter region of myogenin, and we have previously demonstrated that an intact MEF2 site is required for the induction of the myogenin promoter activity by AVP in L6 cells [9]. To establish which regulatory element mediates the inhibitory effect of LY294002 on the AVP-dependent myogenin expression, we transiently transfected L6 cells with a luciferase reporter construct carrying the 84-bp regulatory sequence containing the E1-box, the MEF2 site and the TATA-box of the myogenin promoter (pMyo84-luc). Figure 5A shows that, as expected, after 48 h, the treatment of L6 cells with 0.1 μM AVP increased the pMyo84-luc activity ~4-fold, confirming the presence of an AVP responsive element within this myogenin promoter fragment. However, this effect was dramatically diminished when the cells were treated with both AVP and LY294002, as demonstrated by the ~2.5-fold decrease of pMyo84-luc activity (Figure 5A). We next tested the ability of LY294002 to reduce the AVP-dependent induction of the luciferase activity when the cells were transfected with a construct carrying a mutation in the MEF2 site which prevents MEF2 binding, pMyo84(mMEF2)-luc, or with a construct in which the E1-box site was deleted, pMyo84(-E1)-luc (Figure 5B,C). None of the treatments induced the reporter gene activity in cells transfected with the construct carrying a mutation in the MEF2 site, confirming the crucial role for this factor in the AVP-dependent effect on myogenesis stimulation. In contrast, the deletion of the E1-box did not affect the ability of LY294002 to significantly decrease the AVP-dependent activation of the 84-bp fragment of the myogenin promoter. These results highlight the importance of the PI3K/Akt signaling as a critical player in the molecular mechanism by which AVP can exert its full effect in the enhancement of the myogenic program, via myogenin.

### 2.7. LY294002 Regulates MEF2 Expression and Cellular Localization in AVP-Treated L6 Cells

**Figure 6:**
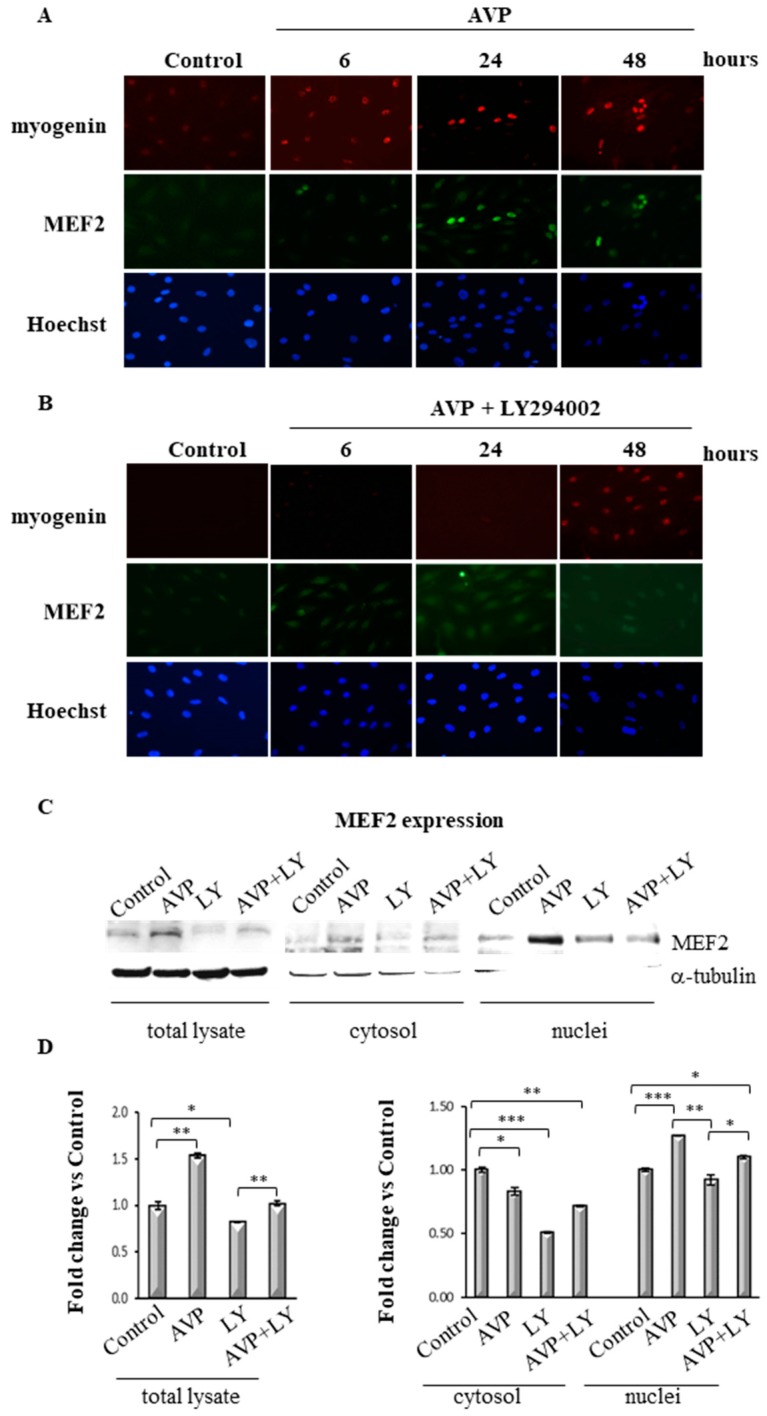
Myogenin and MEF2 expression/cellular localization are modulated by LY294002 in AVP-treated L6 cells. L6 cells were cultured in growth medium and after 24 h were shifted in serum-free medium and treated with 0.1 μM AVP in the presence and in the absence of 20 μM LY294002 for 48 h. (**A**,**B**) Immunofluorescence analysis was performed to evaluate the expression levels and cellular localization of myogenin and MEF2. (original magnification ×20). (**C**) Western blot analyses of total lysates and cytosolic and nuclear fractions were achieved to evaluate changes of cellular localization of MEF2 after LY294002 addition in AVP-treated and un-treated L6 cells. (**D**) Densitometric analyses were performed using an anti-α-tubulin antibody to verify the equal loading of the samples and the cytoplasmic contamination of the nuclear fractions. Nuclear and cytosolic fractions were normalized using Stain-free technology. Representative blots are shown. Statistical analysis was performed by Student’s *t*-test on data obtained from three independent experiments. * *p* < 0.05; ** *p* < 0.01; *** *p* < 0.001.

Based on the above data, we verified whether the inhibition of the PI3K signaling interferes with the AVP-dependent expression and cellular localization of specific relevant transcription factors. To validate this hypothesis, we first investigated, by immunofluorescence analysis, whether the inhibition of the PI3K pathway resulted in the modulation of MEF2 expression and/or cellular localization, and of myogenin expression. For this purpose, we treated L6 cells, cultured in serum-free medium, with 0.1 μM AVP, with or without 20 μM LY294002, and evaluated the expression of myogenin and MEF2 at different time points (6, 24 and 48 h). Figure 6A shows that after 6 h of AVP treatment, both factors were only slightly detectable in the nuclei of L6 cells and that their expression sharply increased at the following time points. At 48 h, indeed, MEF2 and myogenin co-localized in the same subset of nuclei that show a characteristic pattern, forming nuclear rings, a morphological marker of maturation [53]. In contrast, when we treated the cells with the combination of AVP and LY294002, the expression of MEF2 and myogenin was barely detectable at all time points. In particular, MEF2 displayed a very light and more diffuse pattern compared with what observed after AVP-only treatment, its expression being detectable in both the nuclear and the cytoplasmic compartment (Figure 6B). This result suggests that the PI3K is involved in the modulation of the early phases of the AVP-induced myogenic differentiation, since the expression of myogenin is already affected 6 h after the inhibitor addition and, at the same time, the nuclear translocation of MEF2 is prevented. The cellular localization of MEF2 was further confirmed by Western blot assay performed using the total lysates and the cytosolic and nuclear fractions of L6 cells harvested after 48 h of AVP and/or LY294002 addition. As shown in Figure 6C,D, MEF2 was highly expressed in the total lysate of AVP-treated cells, and its expression decreased after the addition of LY294002. In particular, when the cells were treated with AVP alone or in combination with LY294002, MEF2 expression decreased in the cytosolic fraction and increased in the nuclei of L6 cells. These results confirmed the data obtained by immunofluorescence analysis and demonstrated that the AVP-dependent activation of PI3K signaling controls MEF2 cellular localization.

### 2.8. LY294002 Interferes with the AVP-Dependent Cellular Localization of HDAC4 and NFATc3 in L6 Cells

**Figure 7:**
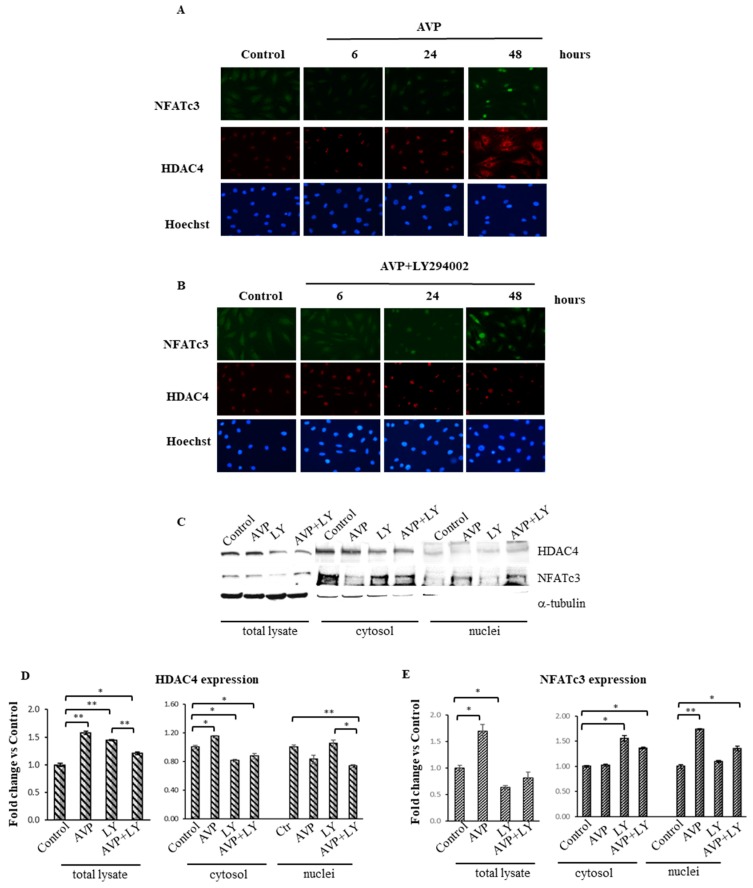
HDAC4 and NFATc3 expression/cellular localization are modulated by LY294002 in AVP-treated L6 cells. L6 cells were cultured in growth medium and after 24 h were shifted in serum-free medium and treated with 0.1 μM AVP in the presence and in the absence of 20 μM LY294002 for 48 h. (**A**,**B**) Immunofluorescences analysis was performed to evaluate the expression levels and cellular localization of HDAC4 and NFATc3 (original magnification 20×). (**C**) Western Blot analyses of total lysates and cytosolic and nuclear fractions were achieved to evaluate changes of cellular localization of HDAC4 and NFATc3 after LY294002 addition in AVP-treated and un-treated L6 cells. (**D**) Densitometric analyses were performed using an anti-α-tubulin antibody to verify the equal loading of the samples and the cytoplasmic contamination of the nuclear fractions. Nuclear and cytosolic fractions were normalized using Stain-Free technology. Representative blots are shown. Statistical analysis was performed by Student’s *t*-test on data obtained from three independent experiments. * *p* < 0.05; ** *p* < 0.01.

Histone deacetylases play a crucial role in the regulation of MEF2 expression [54]. MEF2 was shown to interact with class II HDACs directly, ultimately controlling the transcriptional activity of muscle-specific genes. Moreover, our previous works revealed that in L6 cells, AVP treatment induced the cytosolic compartmentalization of HDAC4 and the recruitment of multifactorial complexes, including MEF2 and Nuclear Factor of Activated T-cells (NFAT), on the MEF2 binding site of the myogenin promoter [9]. To investigate the possibility that the PI3K-Akt pathway may control the AVP-dependent subcellular localization of HDAC4, we performed immunofluorescence analysis of L6 cells treated with AVP in the presence or the absence of LY294002 for 6, 24 and 48 h. HDAC4 was only weakly expressed in control samples (Figure 7A); its expression increased in the nuclei of the cells after 6 and 24 h of AVP treatment and, in correspondence of 48 h, its expression was sharply increased in the cytoplasm, and a modest expression level was detected in the nuclei of L6 cells. The presence of the transcription factor NFATc3 was low and diffused in controls and at the earlier time points, but after 48 h its expression significantly increased in the nuclei of the cells treated with AVP alone (Figure 7A). Interestingly, when AVP was administered in combination with LY294002, HDAC4 expression was restricted to the nuclei. In the same conditions, NFATc3 showed a weak and diffuse pattern at the earlier time point, while its expression is slightly detectable in the nuclei at 24 and 48 h (Figure 7B). Western blotting of cytosolic and nuclear fractions (Figure 7C–E) of L6 cells treated as described above for 48 h, confirmed the data obtained by immunofluorescence, demonstrating that LY294002 decreased the AVP-dependent nuclear accumulation of NFATc3 and the cytoplasmic translocation of HDAC4.

## 3. Discussion

This study aimed to investigate whether the PI3K/Akt signaling is involved in the AVP-dependent myogenic differentiation. We previously reported that AVP enhances skeletal muscle differentiation in both in vitro and in vivo experimental models [3,9,19,22]. We demonstrated that, by interacting with V1a receptors, AVP increases intracellular Ca^2+^ concentration, activating CaMK and calcineurin signaling pathways [18]. Although the inhibition of CaMK and calcineurin pathway decreased the positive effect of AVP on myogenic differentiation, it did not abrogate its action, suggesting that other signaling pathways may be involved in this effect. A role of PI3K in the response of myogenic cells to AVP is suggested by the significant InsP3 generation following stimulation [29] and by the hypertrophic changes of the fibers occurring as a result of the AVP treatment both in culture and in vivo 3, 9, 19, 21, 22]. Interestingly, it was demonstrated that PI3K mediates the stimulatory effect of anabolic signals such as those exerted by Insulin-like Growth Factor IGFs on muscle differentiation [40,55,56,57,58,59] and that the interference of the PI3K activity abolishes this effect. In addition, since it is known that in several systems, such as normal and neoplastic B-lymphoid cells, the Akt/mTOR signaling is stimulated by the Ca^2+^-dependent activation of the CaMK pathway [42,43], it is tempting to speculate an indirect link between Akt/mTOR pathway and AVP signaling as a consequence of AVP-dependent activation of CaMK pathway. We used both pharmacological and genetic approaches to achieve the inhibition of the PI3K/Akt signaling, in the presence and the absence of AVP. All experiments were conducted culturing the cells in a chemically defined medium to avoid interferences by serum factors [19]. In particular, we used LY294002 as a pharmacological inhibitor of PI3K and demonstrated that it caused a dose-dependent decreasing of the AVP-induced differentiation of L6 cells, both in terms of fusion and CK activity (Figure 1 and data not shown).

The role of the PI3K/Akt pathway in the AVP-dependent myogenic differentiation was confirmed analyzing the expression of several molecules known to be involved in the PI3K signaling pathway. In particular, here we show the involvement of not only PI3K but also of its downstream effectors, phospho-Akt and phospho-mTOR, demonstrating the activation of the canonical PI3K/Akt pathway in AVP-stimulated myogenic cells. Consistently, the pharmacological inhibition of PI3K resulted in the inhibition of its downstream effectors, Akt and mTOR, while upregulating the expression of the muscle-specific ubiquitin ligase atrogin-1.

LY294002 is a very frequently used protein kinase inhibitor, employed in cell-based assays due to its stability in aqueous solution, compared to other compounds such as wortmannin [60]. However, since particular caution is needed in using small molecule inhibitors of protein kinases such as LY294002 to exclude any non-specific effect and assess the physiological roles of these enzymes [60], we decided to corroborate the role of Akt as a key player in the myogenic effect of AVP by using RNA interference technique. We showed that the knockdown of Akt expression abrogated the AVP-dependent stimulation of mTOR expression and stimulated the protein catabolism through FoxO transcription factor.

MRFs act in different phases of skeletal muscle differentiation, regulating a specific program of muscle gene expression in temporally and spatially distinct patterns [6]. Among them, myogenin and MRF4 control the later stages of differentiation favoring the formation of mature myofibers and promoting the transcriptional activation of skeletal muscle structural genes. Here we show that the AVP-dependent stimulation of L6 cell differentiation (evaluated by the expression of myogenin and MRF4, fusion index, CK activity, and MHC expression levels), was severely compromised by the simultaneous treatment with LY294002. Such evidence confirms the hypothesis that the PI3K/Akt signaling controls the AVP-dependent myogenic differentiation of L6 cells. These data are in keeping with our previous data demonstrating the central role of InsP3 signaling (very rapid Ins P3 generation, cytosolic Ca^2+^ increase, activation of CaMK) in the AVP stimulation of L6 cells [18,28,29].

Our previous works highlighted a key role for MEF2 in the pro-myogenic effect of AVP, showing that its expression is modulated by AVP treatment and its binding on the myogenin promoter is required for the full expression of the differentiated phenotype. Here we provide evidence that LY294002 hampered the ability of AVP to increase the expression of MEF2 and myogenin, and that this effect was detectable at early time points of AVP treatment. Co-treatment with LY294002 reduced the AVP-dependent stimulation of the pMyo84-luc activity and of the activity of the same E-box-deleted construct. None of the treatments was able to induce the reporter luciferase activity when the cells were transfected with the construct carrying a mutation in the MEF2 site, confirming the crucial role for this factor in the AVP-mediated effect on myogenesis stimulation. Altogether, these results highlight the importance of the PI3K-Akt signaling as a critical player in the molecular mechanism by which AVP can exert its full effect in the enhancement of the myogenic program.

Growing evidence indicates that chromatin-modifying enzymes and remodeling complexes epigenetically reprogram muscle promoters at various steps in many cellular processes, thus modulating MRFs and MEF2 activity. Among these, histone deacetylases (HDACs) and histone acetyltransferases (HATs) alter chromatin structure through post-translational modifications, impacting on MRFs and MEF2 transcriptional activities. Previous data from our group showed that the AVP-mediated myogenic effect is dependent on the nuclear export of HDAC4 through the stimulation of the CaMK and calcineurin signaling pathways [9], leading to the recruitment of multifactorial complexes on the MEF2 binding site and the activation of the myogenin promoter. However, the pharmacological inhibition of CaMK and calcineurin pathways did not completely inhibit the AVP-induced myogenic differentiation, suggesting that other pathways might be involved in this effect. Our results showed that the inhibition of the PI3K pathway by LY294002 repressed the AVP-dependent HDAC4 nuclear export and, consequently, hampered the binding of multifactorial complexes (which include NFATc3 and MEF2) on the promoter region of muscle-specific genes, impinging on the full development of the differentiation program.

**Figure 8:**
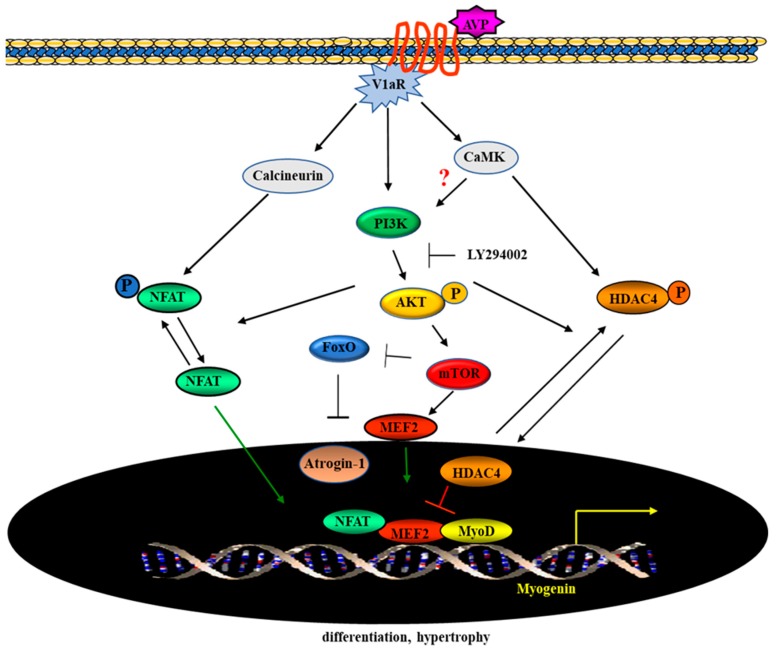
Schematic representation of signaling pathways involved in the AVP-dependent myogenic differentiation. The stimulation of AVP-V1aR signaling in myogenic cells results in the activation of the CaMK and the calcineurin pathways. The AVP-dependent combined activation of both pathways results in the formation of multifactorial complexes on the promoters of muscle-specific genes and strongly stimulates myogenic differentiation. The V1aR is a G-protein coupled receptor, whose occupancy activates PI3K. Inhibition of PI3K/Akt signaling by LY294002 results in mTOR, MEF2, and myogenin downregulation, affects the cellular localization of MEF2 preventing its nuclear translocation, upregulates the expression of FoxO3a leading to the activation of ubiquitin ligase atrogin-1, thus impinging upon the AVP-dependent myogenic differentiation. Additionally, the inhibition of the PI3K pathway by LY294002 represses the AVP-dependent HDAC4 nuclear export and, consequently, hampers the binding of multifactorial complexes (which include NFATc3 and MEF2) on the promoter region of muscle-specific genes, impinging on the full development of the differentiation program. Of note, recent evidence highlights that the Ca^2+^-dependent activation of the CaMK pathway stimulates Akt/mTOR signaling in normal and neoplastic B-lymphoid cells, suggesting a crosstalk between these signaling which may potentiate the myogenic effect of AVP (T-bars represent inhibition).

In conclusion, we provided evidence that the activation of the PI3K/Akt pathway is essential for the full effect of AVP on myogenic differentiation. Using a genetic approach, we demonstrated that AVP controls myogenesis by promoting the activity of mTOR, on the one hand, and by downregulating FoxO transcription factor on the other. Moreover, our results showed that the pharmacological inhibition of the PI3K pathway repressed the AVP-dependent HDAC4 nuclear export, thus precluding the full development of the differentiation program (Figure 8).

It is also interesting to recall that the V1a^-/-^ mouse [61] was reported to show insulin resistance and hyperammonemia, due to increased protein catabolism, suggesting that AVP plays a role in regulating the balance between protein synthesis and degradation. The results reported in this study demonstrate an essential role for the PI3K pathway in the mechanism of the effect of AVP on myogenic differentiation and suggest that AVP, or its downstream signaling, may represent a useful tool to maintain muscle mass and prevent muscle wasting.

## 4. Methods

### 4.1. Cell Cultures

L6 rat myogenic cells, subclone C5 (L6-C5) [3,19], were routinely seeded at the density of 12,000 cells/cm^2^ in DMEM (EuroClone, Milan, Italy) supplemented with 10% Fetal Bovine Serum (FBS) (PAN Biotech, Aidenbach, Germany), 100 units/mL penicillin/streptomycin (Carlo Erba, Milan, Italy) and 10 mM Hepes (PAN Biotech, Aidenbach, Germany). Twenty-four hours after plating, cultures were shifted to a serum-free medium consisting of DMEM supplemented with 1% fatty acid-free Bovine Serum Albumin (Merk, Darmstadt, Germany) [19]. The cells were treated with 0.1 μM AVP (Sigma, St. Louis, MO, USA) and/or different concentration of LY294002 (Sigma, St. Louis, MO, USA) for 48 h. When AVP and LY294002 were used in combination, LY294002 was added 30 min before the beginning of AVP treatment. Cell cultures were maintained at 37 °C in a 5% CO_2_ humidified atmosphere.

### 4.2. Morphological Analysis and Measurement of Myoblast Fusion and Growth

After six days of culture in serum-free medium, morphological differentiation of L6 cells was evaluated by May-Grünwald Giemsa staining (Bio Optica, Milan, Italy) according to the manufacturer’s instructions. Cells were considered fused if cytoplasmic continuity and at least three nuclei were present in each myotube. More than 10 randomly selected fields and more than 300 total nuclei were counted for each sample. The ratio between the number of nuclei in myotubes versus the total number of nuclei per microscopic field was expressed as the percentage of fusion. Each experimental point represented in the graphs is the mean ± SD of the counts obtained from three to five independent samples obtained in three separate experiments. Statistical analysis was performed using the Student’s *t*-test.

### 4.3. Real-Time PCR Analysis

Total RNA was prepared from L6 cells using TRIzol^®^ Reagent (Thermo Scientific, Rockford, IL, USA), following the manufacturer’s protocol. RT-PCR was performed using 1 μg of total RNA reverse transcribed using TaqMan^®^ RNA Reverse Transcription Kit (Applied Biosystem, Foster City, CA, USA). For Real-Time PCR, cDNA synthesis was performed from three independent RNA preparations, whereas quantitative PCR was performed with a Step One Real-Time PCR System instrument (Thermo Scientific, Rockford, IL, USA). PCR reactions were performed using TaqMan gene expression assay for MEF2C: Assay ID Mm00600423_m1; Myf6 (MRF4): Assay ID Mm00435126_m1; Fbxo32 (atrogin-1): Mm01207878_m1 and Hprt: Assay ID Mm00446968_m1 as endogenous control.

### 4.4. Western Blot Analysis

For immunoblotting analysis, cells were washed in 1× PBS, harvested and lysed in 1x Cell Lysis Buffer (Cell Signaling, Danvers, MA, USA) containing 1mM PMSF (Cell Signaling, Danvers, MA, USA) and a complete protease inhibitor cocktail (Cell Signaling, Danvers, MA, USA) for 30 min at 4 °C. Then the cells were briefly sonicated, and the total extracts were centrifuged 10 min at 14,000× *g* in a refrigerated microfuge. Cytoplasmic and nuclear extracts were obtained with NE-PER Nuclear and Cytoplasmic Extraction Reagents kit (Thermo Scientific, Rockford, IL, USA) according to the manufacturer’s instructions. Protein concentration was determined by Bradford Protein Assay (Bio-Rad Laboratories Inc., Hercules, CA, USA), according to the manufacturer’s instructions. Equal amounts of proteins were then separated by SDS/PAGE (4–20% Mini-PROTEAN TGX^TM^ Precast Gels, Bio-Rad Laboratories Inc., Hercules, CA, USA) and transferred to a nitrocellulose membrane (GE Healthcare, Piscataway, NJ, USA). Membranes were blocked with Tris-buffered saline (TBS) 1× (Bio-Rad Laboratories Inc., Hercules, CA, USA) supplemented with 0.1% Tween-20 (Sigma-Aldrich, St. Louis, MO, USA) and containing 5% nonfat milk for 1 h at room temperature (RT). The primary antibodies used in this work were: anti-myogenin (rabbit polyclonal antibody), anti-HDAC4 (rabbit monoclonal antibody), anti-NFATc3 (mouse monoclonal antibody), anti-MEF-2C (mouse monoclonal antibody), all from Santa Cruz Biotechnology, Dallas, TX, USA. Anti-Myosin Heavy Chain (MHC) antibody MF20 (mouse monoclonal antibody, Developmental Hybridoma-Bank, University of Iowa, Iowa City, IA, USA); anti FOXO3a/FKHRL1 (rabbit polyclonal antibody), Merck Millipore; anti-mTOR (rabbit polyclonal antibody), anti phospho-mTOR (rabbit polyclonal antibody), anti-Akt (rabbit polyclonal antibody), phospho-Akt (Thr308) (rabbit monoclonal antibody), anti-p44/42 MAPK (ERK1/2) (rabbit monoclonal antibody), and anti-phospho-p44/42 MAPK (ERK1/2) (Thr202/Tyr 204) (rabbit monoclonal antibody), were from Cell Signaling, Danvers, MA, USA. Anti α-Tubulin (mouse monoclonal antibody, clone B512), Sigma-Aldrich, St. Louis, MO, USA) or anti-β-actin (mouse monoclonal antibody), Sigma-Aldrich were used as a loading control.

Blots were then incubated with horseradish peroxidase-conjugated secondary antibody (Vector Laboratories, Burlingame, CA, USA) for 1 h at RT. Signals were captured by ChemiDoc^TM^ Imaging System (Bio-Rad Laboratories Inc., Hercules, CA, USA) using the Clarity Western ECL enhanced chemiluminescence system (Bio-Rad Laboratories) and densitometric analysis was performed with Image Lab^TM^ Touch Software (Bio-Rad Laboratories). All experiments were carried out in triplicate, and representative results are shown.

### 4.5. Immunofluorescence ASSAY

Immunofluorescence analysis was performed on L6 seeded at the density of 12,000 cells/cm^2^ in 6-well plates on round coverslips in serum-free medium and treated with 0.1 µM AVP alone or in association with 20 µM LY294002 for the time specified in the text. Then they were fixed in cold methanol/acetone (1:1) for 20 min at −20 °C, permeabilized with 1x phosphate buffered saline (PBS) and 0.01% Triton X-100 for 3 min at RT and blocked with 1x PBS, 5% Normal Donkey Serum (Sigma-Aldrich, St. Louis, MO, USA) and 0.3% Triton X-100 for 1 h at RT. The cells were incubated overnight at 4 °C with the following primary antibodies: anti-MHC MF20 antibody (Developmental Hybridoma-Bank University of Iowa, Iowa City, IA, USA); anti-myogenin, anti- NFATc3, anti- MEF-2C, anti-HDAC4 antibodies were from Santa Cruz Biotechnology, Dallas, TX, USA; anti-FoxO3a, from Millipore, Burlington, MA, USA. The next day, the slides were incubated with the following secondary antibodies for 1 h at RT: Alexa Fluor 594 and Alexa Fluor 488 (both 1:1000, Invitrogen Molecular Probes, Eugene, OR, USA).

### 4.6. Transient Transfection of L6 CELLS

L6 cells were plated in 6-well 35-mm culture dishes at a density of 2.5 × 10^5^ cells/well. Twenty-four hours later, cells were transfected with 1.5 µg of expression vectors driven by the myogenin promoter fragments: pMyo84-luc, pMyo84(mMEF2)-luc and pMyo84(-E1)-luc using the Lipofectamine reagent (Thermo Scientific, Rockford, IL, USA) following the manufacturer’s instructions. The plasmid encoding β-galactosidase under the control of the cytomegalovirus (CMV) promoter, CMV-βgal, was included to monitor transfection efficiency and for normalization of reactions. At 24 h after the start of transfection, the transfection mixture was removed and replaced with DMEM + 1% bovine serum albumin and the cells were treated with or without 0.1 μM AVP, 20 μM LY294002 and AVP + LY294002 for 48 h. Cells were then washed twice with PBS and scraped in 1× reporter lysis buffer (Promega, Milan, Italy). Luciferase activity was determined with a luciferase assay kit (Promega, Milan, Italy).

### 4.7. siRNA Transfection

To silence the Akt gene, we used the Akt1 TriFECTa Kit DsiRNA Duplex (IDT Tech., Inc. Coralville, IA, USA). L6 myoblasts were plated in six-well plates at the density of 30,000/cm^2^ and cultured as previously reported. The cells were transfected using Mirus TransIT-X2^®^ Transfection Reagent (Mirus Bio Corporation, Madison, WI, USA) according to the manufacturer’s instructions. Briefly, 25 nM of siRNAs were diluted in 0,25 mL of Opti-MEM I Reduced Serum Media (Thermo Fisher Scientific, Waltham, MA, USA) and 7.5 μL of Mirus TransIT-X2^®^ Transfection Reagent for each well. The mixture was preincubated at RT for 30 min before adding to the cells. After 48 h, cells were washed with PBS and shifted to DMEM supplemented with 1% BSA and treated as described in the text above. Western blot analysis was performed to determine the efficiency of Akt1 siRNA silencing (Supplemental Figure S1a). TYE 563 Transfection Control DsiRNA was used as a fluorescently labeled transfection control (Supplemental Figure S1b).

### 4.8. Statistical Analysis

Each experiment was repeated three times. Data are presented as the mean ± SD. Statistical analysis was performed using the Student’s *t*-test, assuming equal variance, and *p*-values were calculated based on the 2-tailed test. A *p*-value of ≤0.05 was considered statistically significant.

## Supplementary Materials

Supplementary Materials can be found at https://www.mdpi.com/1422-0067/20/17/4188/s1.

## Availability of Data Material

The datasets used and/or analyzed during the current study are available from the corresponding authors on reasonable request.

## Abbreviations

Akt	Protein kinase B
AVP	Arg^8^-vasopressin
CaMK	Ca^2+^-Calmodulin-dependent protein kinase
CnA	Calcineurin A
FoxO	Forkhead box O transcription factor
HATs	Histone acetyltransferases
HDACs	Histone deacetylases
b-HLH-MRFs	Basic-Helix-Loop-Helix Myogenic Regulatory Factors
InsP3	inositol 1,4,5-trisphosphate
LY294002	2-(4-morpholinyl)-8-phenyl-4H-1-benzopyran-4-one
MEF2	Myocyte Enhancer Factor 2
MHC	Myosin heavy chain
OT	oxytocin
PI3K	Phosphoinositide 3-Kinase
mTOR	Mammalian Target of Rapamycin
V1aR	V1a vasopressin Receptor
