# Supplementary figures and images for: Inhibition of Phosphoinositide 3-Kinase/Protein Kinase B Signaling Hampers the Vasopressin-dependent Stimulation of Myogenic Differentiation
Source: Int J Mol Sci. 2019 Aug 27;20(17):4188. doi: 10.3390/ijms20174188 (PMC6747374; doi:10.3390/ijms20174188)

## Effect of AKT silencing

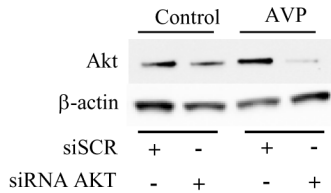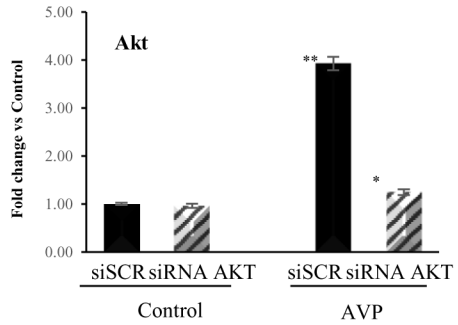

## Transfection efficiency (TYE 563)

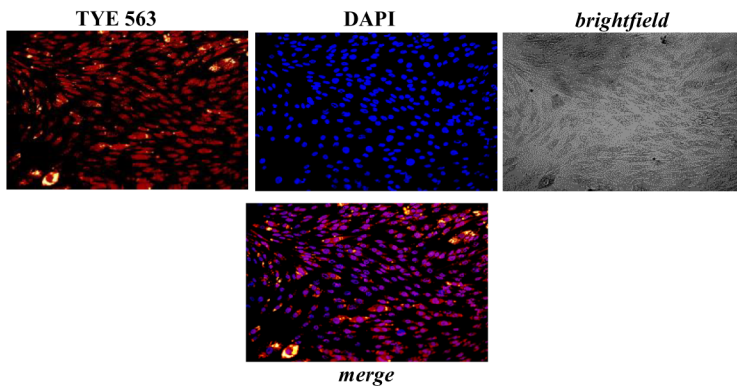

Supplemental Figure 1

Supplement: Supplementary file 1 [file ijms-20-04188-s001.pdf]
